# Next-generation sequencing yields complete mitochondrial genome assembly of peaceful betta fish, *Betta imbellis* (Teleostei: Osphronemidae)

**DOI:** 10.1080/23802359.2020.1841582

**Published:** 2020-12-24

**Authors:** Syed Farhan Ahmad, Nararat Laopichienpong, Worapong Singchat, Aorarat Suntronpong, Tavun Pongsanarm, Thitipong Panthum, Nattakan Ariyaraphong, Jakaphan Bulan, Tanawat Pansrikaew, Kornsuang Jangtarwan, Navapong Subpayakom, Sahabhop Dokkaew, Narongrit Muangmai, Prateep Duengkae, Kornsorn Srikulnath

**Affiliations:** a Department of Genetics, Faculty of Science, Laboratory of Animal Cytogenetics and Comparative Genomics (ACCG), Kasetsart University, Bangkok, Thailand; b Department of Forest Biology, Faculty of Forestry, Special Research Unit for Wildlife Genomics (SRUWG), Kasetsart University, Bangkok, Thailand; c Department of Aquaculture, Faculty of Fisheries, Kasetsart University, Bangkok, Thailand; d Department of Fishery Biology, Faculty of Fisheries, Kasetsart University, Bangkok, Thailand; e Center for Advanced Studies in Tropical Natural Resources, National Research University-Kasetsart University (CASTNAR, NRU-KU), Kasetsart University, Bangkok, Thailand; f Center of Excellence on Agricultural Biotechnology (AG-BIO/PERDO-CHE), Bangkok, Thailand; g Amphibian Research Center, Hiroshima University, Higashihiroshima, Japan

**Keywords:** Peaceful betta, bubble-nesting fighting fish, bioresource

## Abstract

The complete mitochondrial genome (mitogenome) of the peaceful betta (*Betta imbellis*) was obtained using next-generation sequencing. The sample of *B. imbellis* was collected from its native habitat in Southern Thailand. The mitogenome sequence was 16,897 bp in length, containing 37 genes with identical order to most teleost mitogenomes. Overall nucleotide base composition of the complete mitogenome was determined as AT bias. Phylogenetic analysis of *B. imbellis* showed a closer relationship with bubble-nesting fighting fish. This annotated mitogenome reference can be utilized as a bioresource for phylogenetic studies to support betta conservation programs.

Southeast Asia (SEA) is a well-known biocultural hotspot of biodiversity and endemism encompassing a huge variety of betta fish (*Betta* spp.) belonging to the family Osphronemidae (Panijpan et al. 2014). The bubble-nesting species *Betta imbellis*, also known as the peaceful betta, is native to SEA and one of the most popular wild type betta fish for fishkeeping hobbyists (Kusrini et al. 2015). This betta fish inhabits still and sluggish waters including rice paddies, swamps, roadside ditches, streams, and ponds. Artificial selection activities for ornamental and aquarium trade purposes have resulted in inbreeding and outbreeding depression with an adverse effect on the genetic integrity of wild populations (Beer et al. 2019). Here, a complete mitochondrial genome (mitogenome) of *B. imbellis* collected from Nakhon Si Thammarat, Thailand (8.4325° N, 99.9599° E), was assembled, annotated, analyzed, and stored in the Thailand Natural History Museum (no. THM21221). Whole genomic DNA was extracted in accordance with the standard salting-out protocol (Supikamolseni et al. 2015), and next-generation sequencing was performed using an Illumina HiSeq platform at Vishuo Biomedical (Thailand) Ltd. (Bangkok, Thailand). Quality of Illumina reads was evaluated with FastQC software and the raw reads were trimmed to discard adapters using Trimmomatic (Bolger et al. 2014). The trimmed reads were subjected to alignments to isolate all mitogenome sequences by mapping whole genome Illumina reads against the complete mitogenome of *B. splendens* (AB571120), using bwa default parameters (Li and Durbin 2009). The mapped alignment was processed using Samtools (Li et al. 2009), and aligned reads were extracted using Bedtools (Quinlan and Hall 2010). Aligned reads with the mitogenome were then de novo assembled using Velvet (Velvet_1.1.07; kmer = 123) (Zerbino and Birney 2008). A total of 395,074 individual reads gave a mean coverage of more than 330X for the generated contigs. We then assembled a consensus scaffold of the complete mitogenome using the reference-based assembly approach in Geneious Prime (https://www.geneious.com/prime/), by mapping the Velvet contigs against the reference mitogenome of *B. splendens.* The assembled mitogenome was annotated in the MITOS WebServer (Bernt et al. 2013). Complete mitogenome sequences consisted of 16,897 bp for *B. imbellis* (GenBank Accession number: MT988019, SRA: SRR12614920, BioProject: PRJNA662470), containing 37 genes and a control region (CR). Gene arrangement patterns were identical to those of teleosts (Miya et al. 2013). Overall AT content values for the mitogenome were 61.9%. Average nucleotide diversity among all *Betta* mitogenomes was determined at 24.56 ± 6.04%. Four conserved sequence blocks (CSB-D, CSB1, CSB2, and CSB3) in the CR of teleost mitogenomes were also present in *B. imbellis* (Lee and Kocher 1995; Prakhongcheep et al. 2018; Ponjarat et al. 2019; Singchat et al. 2020). Diverse numbers of tandem repeats were observed among *Betta* species (Song et al. 2016; Prakhongcheep et al. 2018; Ponjarat et al. 2019; Singchat et al. 2020), suggesting that the CR had large variation in different fighting fish species. A phylogenetic tree was constructed based on 12 concatenated protein-coding genes without *ND6* of 17 teleosts, using Bayesian inference with MrBayes version 3.2.6 (Huelsenbeck and Ronquist 2001). The group comprising *B. splendens*, *B. mahachaiensis*, *B. smaragdina*, and *B. imbellis* formed a monophyletic clade as bubble-nesting fighting fish, consistent with Ruber et al. (2004), Sriwattanarothai et al. (2010), and Singchat et al. (2020) (Figure 1). These complete mitogenomes comprise a reference annotated genome, and provide valuable information at the molecular level that can be utilized to sustain betta bioresources to improve conservation programs and commercial breeding management.

**Figure 1.**
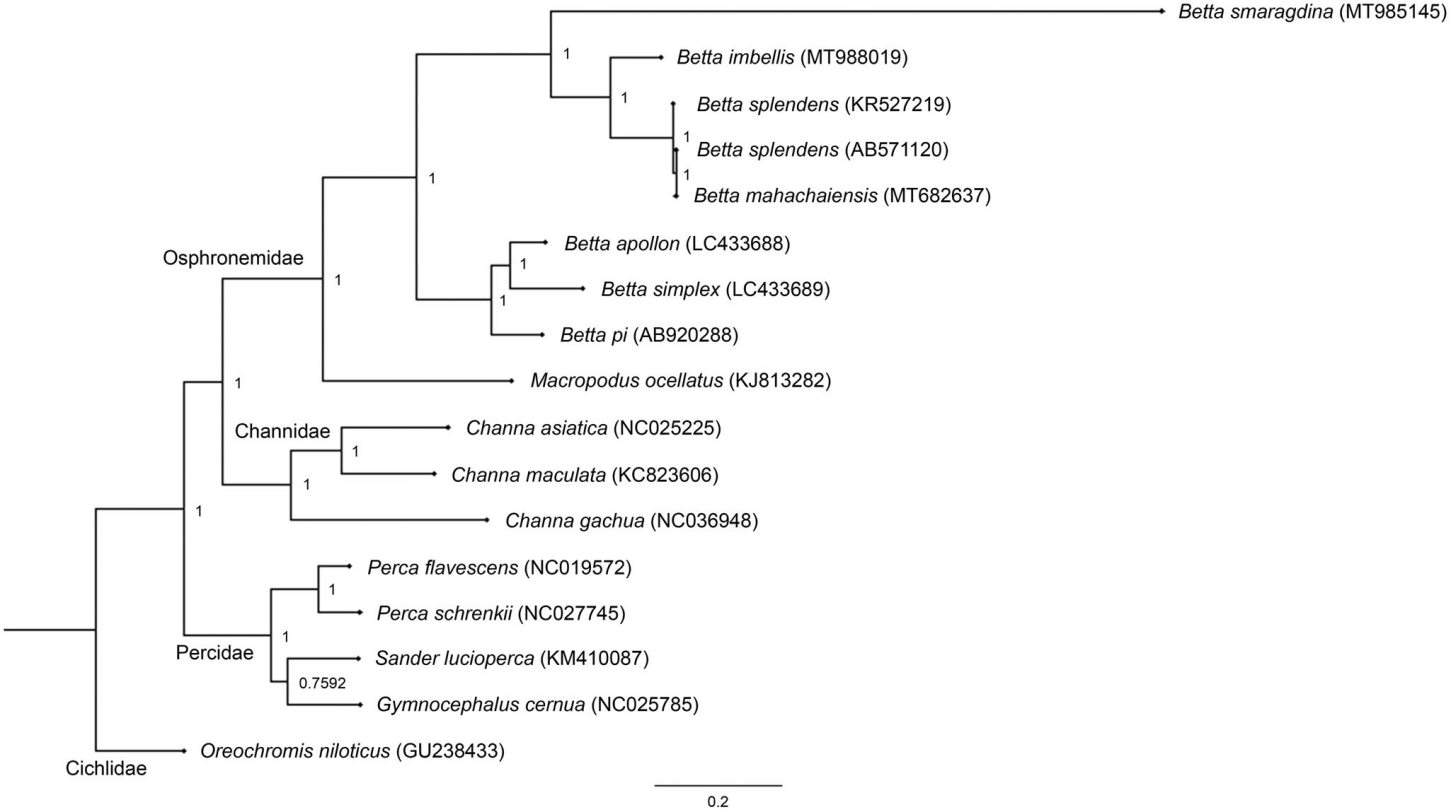
Phylogenetic relationships among 12 concatenated mitochondrial protein-coding genes, without *ND6* sequences of 17 mitochondrial genomes including *Oreochromis niloticus* as the outgroup, using Bayesian inference analysis. The complete mitochondrial genome sequence was downloaded from GenBank. Accession numbers are indicated in parentheses after the scientific names of each species. Support values at each node are Bayesian posterior probabilities, while branch lengths represent the number of nucleotide substitutions per site.

## Data Availability

Data supporting the findings of this study are openly available in SRA and GenBank of NCBI at https://www.ncbi.nlm.nih.gov. The isolated mitogenome reads were deposited at the NCBI SRA database (accession ID: SRR12614920), and assembled mitogenome sequences are available in GenBank (accession ID: MT988019) under the BioProject: PRJNA662470.
